# Self-Identified Stage in Recovery and Substance-Use Behaviors among Pregnant and Postpartum Women and People with Opioid Use Disorder

**DOI:** 10.3390/healthcare11172392

**Published:** 2023-08-25

**Authors:** Hannah S. Szlyk, Anna Constantino-Pettit, Xiao Li, Erin Kasson, Emily Maranets, Yoseph Worku, Mandy Montayne, Devin E. Banks, Jeannie C. Kelly, Patricia A. Cavazos-Rehg

**Affiliations:** 1 Department of Psychiatry, Washington University School of Medicine, 660 South Euclid Avenue, St. Louis, MO 63110, USA; aconstantino@wustl.edu (A.C.-P.); lixiao@wustl.edu (X.L.); erinmkasson@wustl.edu (E.K.); e.maranets@wustl.edu (E.M.); w.yoseph@wustl.edu (Y.W.); mamanda@wustl.edu (M.M.); pcavazos@wustl.edu (P.A.C.-R.); 2 Brown School, Washington University in St. Louis, 1 Brookings Dr., St. Louis, MO 63130, USA; 3 Department of Psychological Sciences, University of Missouri–St. Louis, One University Blvd., 325 Stadler Hall, St. Louis, MO 63121, USA; devinbanks@umsl.edu; 4 Department of Obstetrics & Gynecology, Washington University School of Medicine, 660 S. Euclid Ave., St. Louis, MO 63110, USA; jckelly@wustl.edu

**Keywords:** maternal healthcare, pregnancy, postpartum, opioid use disorder, stage in recovery

## Abstract

Opioid use among pregnant and postpartum women and people (PPWP) has significant health repercussions. This study explores how substance-use behaviors may vary by stage in recovery among PPWP with opioid use disorder (OUD). We recruited 29 PPWP with OUD. “High-risk” participants self-identified as “*not being engaged in treatment*” or “*new or early in their recovery*” (n = 11); “low-risk” participants self-identified as being “*well-established*” or “*in long-term recovery*” (n = 18). Participants were queried regarding sociodemographic, mental health, and drug-misuse factors; urine drug screens were collected at baseline. Univariate group comparisons between high-risk and low-risk PPWP were conducted. High-risk PPWP were more likely to self-identify as non-Hispanic African American and more likely to report current opioid use, other illicit drugs, and tobacco. High-risk PPWP had higher opioid cravings versus low-risk PPWP. High-risk PPWP were more likely to screen positive on urine tests for non-opioid drugs and on concurrent use of both non-opioid drugs and opioids versus low-risk participants. PPWP earlier in recovery are at higher-risk for opioid and other illicit drug misuse but are willing to disclose aspects of their recent use. PPWP early in recovery are an ideal population for interventions that can help facilitate recovery during the perinatal period and beyond.

## 1. Introduction

Opioid Use Disorder (OUD) is characterized by a problematic pattern of opioid use leading to clinically significant functional impairment or distress [1]. Globally, over 16 million people would meet criteria for OUD [2]. In the United States (U.S), it is estimated that 6.7 to 7.6 million people are currently living with OUD [3]. Although attempts have been made to mitigate the known risks of OUD among pregnant and postpartum women and people (PPWP), the incidence of OUD among this population in the U.S. has increased continuously. According to a review of U.S. delivery reports from 2010 to 2017, there was a 131% increase in the number of people with an opioid-related diagnosis during delivery, and the incidence of babies born with neonatal opioid withdrawal syndrome (NOWS) increased by 82% during the same period [4]. Untreated OUD during pregnancy has also been associated with several negative consequences for both postpartum individuals and babies, including preterm birth, low birthweight, and difficulty breastfeeding [5,6]. Postpartum individuals specifically have a higher risk of recurrence of opioid use, overdose, and death [7].

Overall, pregnancy and postpartum are crucial stages to address opioid misuse and other substance misuse among diverse subgroups. The outcomes of PPWP with OUD who receive MOUD are more favorable, with higher birth weights among infants, fewer instances of preterm birth, shorter hospital stays, sustained MOUD treatment, and a greater retention of custody of children [8]. Continued adherence to MOUD in the postpartum period has been associated with fewer recurrences of OUD [9] and a decreased risk of overdose [10]. Thus, it is important to engage PPWP in OUD treatment, including MOUD, as soon as possible.

Moreover, it is important to consider the challenges that individuals face in different stages in recovery and how that impacts both recovery behaviors and substance-use behaviors. Individuals who are in the first few months of recovery from opioids may be at greater risk of recurrence of use than individuals in later stages of recovery and who had misused non-opioid substances [11]. Some reasons for the early recurrence of opioid misuse include less developed coping skills, intense cravings, and polysubstance use [12]. Thus, a person’s stage in recovery may be indicative of level of risk for the recurrence of use and overdose. Yet, less is known about how substance-use behaviors may vary by stage in recovery among PPWP with OUD.

The current study addresses this gap in the literature by utilizing quantitative, qualitative, and electronic health care records to illuminate how substance-use behaviors may vary by self-perceived stage in recovery among PPWP with opioid use disorder (OUD). Mixed-method approaches can provide a more accurate view of research topics by integrating multidimensional insights of a person’s recovery experience [13]. These approaches are particularly suited for capturing the experiences of populations that are underserved, such as PPWP. Study findings have implications for how providers can better support PPWP at higher risk of recurrent use, especially those who belong to minoritized and underserved populations.

## 2. Materials and Methods

### 2.1. Participants and Procedures

The present study analyzed data among participants who were recruited from a clinic that primarily supports PPWP with OUD. The clinic patients were provided with prenatal care and opiate addiction treatment (medication + counseling), and were closely supervised (e.g., weekly visits, urine drug screens, counseling session attendance) by the clinic medical team. Between March 2018 to March 2021, a total of 66 PPWP were recruited to participate in a separate mobile health trial for one-month.

Study eligibility included having a diagnosis of OUD while being currently pregnant or one year postpartum, 18 years or older, a U.S. resident, fluent in English, and owning a smartphone with an iOS or Android operating system. Preliminary outcomes demonstrated that PPWP *uMAT-R* clients represented a diverse sample with specific needs and challenges for recovery (e.g., one third identified as Black/African American and the majority had completed less formal education than a high school diploma/GED). Participants who engaged with their coach were less likely to crave opioids and they reported having more of their basic needs met versus those with no interaction with their coach.

Research staff reviewed the informed consent document with eligible participants via phone or in-person, and participants provided verbal or written consent to join the study. This study was conducted in accordance with the Declaration of Helsinki and was approved by the Washington University Institutional Review Board (IRB #201805132). The EPIC medical chart data were obtained for participants’ demographics, current diagnoses, urine drug screens, education level, insurance type, if the father of the baby (FOB) was involved, stability of their current living situation, number of live births, and whether the pregnancy was planned or not. EPIC data were pulled and coded from December 2020–January 2021. The research team followed IRB protocol to ensure the safety, confidentiality, and privacy of the study participants.

A total of 30 PPWP completed the qualitative interview questions via the secure online platform, REDCap, after two months of access to the *uMAT-R* intervention. Among them, 29 PPWP responded to the item assessing self-identification of the recovery process from the qualitative interview: “Think about your recovery outside of your facility, at what stage in your recovery would you consider yourself to be”. These 29 were included as the final study sample.

### 2.2. Measures

**Self-perceived stage of recovery and risk level (low vs. high).** During the interview, each participant answered the following question: “Think about your recovery outside of your facility, at what stage in your recovery would you consider yourself to be:”. Response options included (1) not engaged in treatment or a recovery program; (2) new to recovery; (3) early in recovery; (4) well-established or long-term recovery. We collapsed PPWP with OUD into two groups depending on their self-identified stage of recovery. “High-risk” participants self-identified as “not being engaged in treatment” or “new or early in their recovery” and “low risk” participants self-identified as being “well-established or in long-term recovery”.

### 2.3. Data from EPIC System

**Socio-demographics.** Demographic information was obtained from the client’s EPIC medical chart. The following items were collected: age (18–24 vs. 25–34 vs. ≥35 years old), self-identified race (White vs. African Americans), pregnancy status (1st or 2nd trimester vs. 3rd trimester or delivered), marital status (married, single, other), type of medication for addiction treatment (buprenorphine/Subutex) vs. other (i.e., methadone), education level (high school diploma/GED or below vs. college or above), insurance (private, Medicaid, other), number of live births (0–1 vs. ≥2), housing (stable vs. unstable (e.g., homeless or living in medical centers/treatment facilities)), employment (yes (part-time job included) vs. no), father of baby involvement (yes vs. no), and planned pregnancy (planned vs. unplanned).

**Lab results from urine drug screen.** At each medical appointment, participants were routinely asked to provide a urine drug screen (UDS). The testing included the standard 7-panel screen, which includes testing for the following substances: cannabis, cocaine, opiates, PCP, amphetamines, benzodiazepines, and barbiturates. Some participants additionally completed a pain management profile, which tested for the following opiates (MOUD and non-MOUD specifically): 6-acetylmorphine, buprenorphine, codeine, hydrocodone, hydromorphone, methadone, morphine, oxycodone, oxymorphone, tapentadol, and tramadol. All positive results were repeated and verified using mass spectrometry in the hospital laboratory.

**Current diagnoses.** Participants’ current psychiatric, substance use, and medical diagnoses were collected from the EPIC medical chart and diagnoses were based on the International Classification of Diseases, Tenth Revision (ICD-10) (See Appendix A). Diagnoses were coded as binary variables. Psychiatric diagnoses include depression (including peripartum depression), anxiety disorder, and other. Substance-use diagnoses include methamphetamine, tobacco, alcohol, cocaine, cannabinoids, and other. Medical diagnoses were broken up into three groups: chronic medical conditions, obstetric diagnoses (high-risk pregnancy, gestational diabetes, gestational hypertension, and history of obstetrical abnormality), and sexually transmitted infections (STI).

### 2.4. Drug-Related Characteristics from Survey Data

**Past 30 days substance use.** The measure assessed the use of multiple different forms of substances within the past 30 days, including tobacco, cannabis, other illicit drugs, and opioids such as heroin, methadone, morphine, OxyContin, codeine, fentanyl, oxycodone, hydrocodone, hydromorphone, and other opioid analgesics and pain killers. All variables were dichotomized as either yes or no.

**Lifetime overdose.** Overdoses were assessed by asking, “How many times have you overdosed on drugs?” Responses were collapsed into never versus at some point in lifetime.

**Craving scale.** Current craving for opioids was assessed using three items adapted from the Cocaine Craving Scale [14]. For each item, the score ranged from 0 to 10 and possible scale scores ranged from 0 to 30, with higher scores indicating a stronger craving.

### 2.5. Mental Health Characteristics

**Depression.** The 9-item Patient Health Questionaire-9 (PHQ-9) is a screening instrument that measures each of the 9 diagnostic criteria for depression from the Diagnostic and Statistical Manual for Mental Disorders, Fourth Edition (DSM-IV), which is commonly used in the substance-use and mental health literature, and has demonstrated excellent internal validity (e.g., Cronbach’s alpha: 0.84) [15]. Possible scale scores ranged from 0 to 27, with higher scores indicating more severe depressive symptoms. A cutoff of 9 was applied to dichotomize as none or mild vs. moderate or above depression.

**Anxiety**. The General Anxiety Disorder-7 (GAD-7) is a screening instrument that measures anxiety symptom severity based on some of the DSM-IV criteria for General Anxiety Disorder (GAD), widely used in mental health research (Cronbach’s alpha: 0.91) [16]. Item scores ranged from “0” (not at all) to “3” (nearly every day) and possible scale scores ranged from 0 to 21, with higher scores indicating more severe anxiety symptoms. A cutoff of 9 was applied to dichotomize as none or mild vs. moderate or above anxiety.

**Suicide**. Lifetime suicide attempt and suicidal ideation in the prior 30 days were each assessed and dichotomized as present (yes) or absent (no).

### 2.6. Statistical Data Analyses

The summary of questions of interest in the present study are presented in Supplementary Table S1. In order to answer the research question, all PPWP were grouped to either a high-risk (e.g., not engaged in treatment or a recovery program, new or early in recovery) or low-risk group (e.g., well established or long-term recovery), based on their responses to the question assessing self-perceived stage of recovery. Descriptive statistics, including frequency counts and percentages for categorical variables and medians and interquartile ranges for continuous variables, were reported for the entire sample and by self-perceived risk level. Given the non-normal distribution of the data, the *p*-value was calculated using either the CHISQ test or Mann–Whitney U test, depending on the type of the variable. The exact test was conducted if the cell size was not sufficiently large (e.g., smaller than 5).

All the statistical analyses were two-sided, and *p*-values < 0.05 were considered statistically significant. Additionally, *p*-values < 0.10 were considered marginally significant, accounting for the small sample size. All statistical analyses were performed using SAS version 9.4.

## 3. Results

A total of 11 of participants were identified by the research team as “high-risk” and 18 PPW self-identified as “low-risk” (see Table 1). No difference was observed in socio-demographic characteristics, with the exception of the racial group variable. More PPWP in the high-risk group self-identified as Black/African American (high-risk: 50% vs. low-risk: 7%, χ^2^ (1, 26) = 5.06, *p* = 0.04).

Table 2 displays the results of drug-related characteristics and the differences in between groups. Over half of the sample reported at least one past overdose in their lifetime (n = 13, 54.2%.). Significant differences were observed between groups on drug-use variables. Specifically, in the high-risk group, more PPWP reported currently using opioids (high-risk: 60% vs. low-risk: 8%, χ^2^ (1, 23) = 7.30, *p* = 0.02), tobacco (high-risk: 100% vs. low-risk: 46%, χ^2^ (1, 23) = 7.74, *p* = 0.01), and other illicit drugs besides opioids and heroin (high-risk: 50% vs. low-risk: 0%, χ^2^ (1, 22) = 7.76, *p* = 0.01). Additionally, PPWP in the high-risk group had higher rates of craving opioids versus PPWP in the low-risk group (median (IQR), high-risk: 12.0 (4.0, 20.0) vs. low-risk: 1.50 (1.0, 14.0), z = 1.91, *p* = 0.07). Also, high-risk PPWP were more likely to screen positive on urine test results for non-opioid drugs (including barbiturates, benzos, cocaine, cannabis, etc.) (high-risk: 75% vs. low-risk: 25%, χ^2^ (1, 26) = 5.06, *p* = 0.04) and test positive on urine test results for concomitant use of non-opioid drugs plus non-MOUD opioids (oxycodone, heroin, fentanyl, etc.) (high-risk: 70% vs. low-risk: 30%, χ^2^ (1, 26) = 5.11, *p* = 0.04) versus low-risk participants. No difference was observed in mental health comorbidities between the groups (as shown in Table 3).

## 4. Discussion

This study is among the first to directly ascertain PPWP’s self-perceived stage in recovery from opioid- and other substance-use disorders and associate the stage of recovery with substance-use behaviors. We employed a mixed-methods approach to better capture the experiences of an underserved population in recovery. We found that over half of our sample endorsed a history of overdose in their lifetime. This finding emphasized the potential risk among PPWP with substance misuse—regardless of their self-identified stage of recovery. Overall, PPWP who were defined as high risk reported more substance-use behaviors when compared to PPWP at low-risk. Of note, the most used substance across both the high-risk and low-risk groups was cannabis. It is possible that cannabis is being used to manage opioid withdrawal or pain symptoms [17,18]. This finding is important as recent changes in legislation regarding cannabis legalization—including for the area in which the participants for the current study reside—may contribute to elevated use among PPWP. Recent data have shown that prenatal cannabis use has increased in recent years—in contrast to trends in prenatal alcohol and nicotine use [19]. Future research should continue to monitor trends in cannabis use among PPWP, including individuals with OUD, to examine any impacts on the childbearing individual and the baby.

Black/African American PPWP were more likely to self-identify as earlier in recovery compared to White PPWP. This finding aligns with other studies demonstrating that OUD and its consequences may disproportionately impact PPWP who belong to minoritized and underserved groups. Studies have found that Hispanic and non-Hispanic Black PPWP are more affected by OUD-related pregnancy complications such as preterm birth and very preterm birth and are less likely to have access to medication for Opioid Use Disorder (MOUD) when compared with non-Hispanic White PPWP [20,21]. These disparities are likely reflective of the institutional barriers (e.g., fewer prescribers and appropriate providers in predominantly Black/African American neighborhoods) and socioeconomic inequities (e.g., lack of transportation to appointments, insurance coverage, and childcare) that exist for Black/African American PPWP seeking support for substance-use disorders [22]. This problem may also be reflective of Crenshaw’s theory of intersectionality, as it applies to Black/African American PPWP attempting to navigate both the medical system and recovery from opioids in the context of historical (and present) racism in the U.S. [23].

PPWP with substance misuse can be understood as an intergenerational public health concern, as substance misuse can have long-lasting effects for both the PPWP and their infant [24,25,26]. Therefore, understanding the association between self-identified stage in recovery and substance-use behavior is important to devise effective interventions with this population. Overall, our findings emphasize the need to enhance screening, treatment accessibility, and interventions for PPWP with OUD, especially those who belong to racially and ethnically minoritized groups.

### 4.1. Implications for Practice

Providers are charged with the task of creating welcoming, receptive, and empowering environments in which PPWP can receive support for recovery from substance-use disorders. Tools exist to make this a reality, some of which are being employed at the clinics from which these participants were recruited. Improving trauma-informed care, providing doula support, providing educational materials, and co-locating MOUD sites and behavioral health clinics within obstetric clinics are feasible and effective ways of working with PPWP with substance-use disorders. If possible, clinics should employ providers and staff who represent diverse identities and offer community-based events focused on wellness and cultivating trust within the community. These strategies reflect hopeful parameters to build trust and rapport with the treatment team that were seen within this study population. For instance, participant self-reported substance-use behavior corresponded with urine drug screen results. Participants were also willing to disclose their self-identified phase in recovery. PPWP is a population that is deserving of more time and resources, and that can be engaged in treatment [27].

Additionally, PPWP at a higher-risk level of recovery may benefit from greater support in reducing substance use and managing cravings. Clinicians may ask about and encourage PPWP to self-reflect on their stage of recovery. This may include prompts about what this stage may mean to them, especially if/when a client indicates that they are “new” or “early” to recovery. Such prompts may be a less stigmatizing way to identify the clients who need extra support and immediate help. Due to significant health risks for the childbearing person and the baby, harm-reduction approaches may be ways for providers to increase rapport, trust, and build recovery momentum with their patient, especially individuals who are newer to recovery [28]. Also, due to the high potential for overdose among this population [29,30], providers should proactively educate clients about the benefits of MOUD, such as reductions in overdose risk, cravings and mood issues [31,32]. Finally, our finding that low-risk PPWP reported less use of substances, fewer cravings, and had fewer positive urine tests than high-risk PPWP is promising. This suggests that PPWP in later stages of recovery who are engaged with treatment do demonstrate improvements in substance-use and related behaviors.

### 4.2. Limitations

Primarily, this study was limited in its statistical power due to the low sample size. While we performed statistical tests to account for small cell sizes, the low count in some of the groups for our bivariate analyses means that these results should be interpreted conservatively. It will be important to corroborate these findings with a larger cohort. Another limitation of the study was its cross-sectional design. While we found some significant associations in demographic and drug-related differences between high-risk and low-risk PPWP, we are unable to speak to the directionality of any of the associations. Survey responses were based on self-report and, therefore, were subject to bias. However, this project was able to cross-reference self-reported drug use with urine drug screens obtained from the obstetric clinic where participants were recruited, which is a relative strength. Lastly, the study sample was specific to a region of the U.S. and may not be generalizable to other regions or countries.

## 5. Conclusions

This study examined differences between PPWP who self-identified as early or late in their recovery. We found that PPWP who were early in recovery—high-risk PPWP—used more substances during pregnancy and experienced higher cravings when compared to PPWP who were later in recovery. Additionally, among the high-risk group, we found that Black/African American PPWP were more likely to report the recent use of opioids, other illicit drugs, and tobacco. These results reflect a need for increased engagement with PPWP who identify as earlier in recovery. Future research should examine the efficacy and feasibility of co-located programs that could decrease the use of opioids and other drugs during pregnancy for those who are early in recovery.

## Figures and Tables

**Table 1:** Sociodemographic characteristics among the study sample by self-perceived stage of recovery (n = 29).

	All (n = 29)	Low-Risk (n = 18)	High-Risk (n = 11)		*p*
	N (%)			χ^2^	
Age					
18–24 years	3 (11.5)	2 (13.3)	1 (9.1)	2.78	0.42
25–34 years	20 (76.9)	10 (66.7)	10 (90.9)		
>35 years	3 (11.5)	3 (20.0)	NA		
Missing	3	3	NA		
Race					
White	18 (69.2)	13 (86.7)	5 (45.5)	5.06	**0.04**
Black/African American	8 (30.8)	2 (13.3)	6 (54.5)		
Missing	3	3	NA		
Pregnant status					
1st–2nd trimester (week 0–26)	16 (66.7)	9 (64.3)	7 (70.0)	0.09	0.99
3rd trimester to delivered	8 (33.3)	5 (35.7)	3 (30.0)		
Missing	5	4	1		
Marital Status					
Single	22 (84.6)	13 (86.7)	9 (81.8)	0.11	0.99
Married	2 (7.7)	1 (6.7)	1 (9.1)		
Other (divorced, widowed, separated)	2 (7.7)	1 (6.7)	1 (9.1)		
Missing	3	3	NA		
Type of MOUD					
Buprenorphine/Subutex	19 (73.1)	13 (86.7)	6 (54.5)	3.33	0.09
Others	7 (26.9)	2 (13.3)	5 (45.5)		
Missing	3	3	NA		
Education attainment					
High school or below	15 (57.7)	8 (53.3)	7 (63.6)	0.28	0.60
(Some) College or above	11 (42.3)	7 (46.7)	4 (36.4)		
Missing	3	3	NA		
Insurance					
Uninsured	1 (3.6)	0	1 (9.1)	4.83	0.09
Medicaid	26 (92.7)	16 (94.1)	10 (90.9)		
Others	1 (3.6)	1 (5.9)	NA		
Missing	1	1	NA		
Housing					
Unstable	4 (15.4)	1 (6.7)	3 (27.3)	2.07	0.28
Stable	22 (84.6)	14 (93.3)	8 (72.7)		
Missing	3	3	NA		
Employment					
No	20 (76.9)	10 (66.7)	10 (90.9)	2.10	0.20
Yes	6 (23.1)	5 (33.3)	1 (9.1)		
Missing	3	3	NA		
Number of live births					
0–1	18 (69.2)	12 (80.0)	6 (54.5)	1.93	0.22
≥2	8 (30.8)	3 (20.0)	5 (45.5)		
Missing	3	3	NA		
Father of baby involved					
No	5 (19.2)	2 (13.3)	3 (27.3)	0.79	0.62
Yes	21 (80.8)	13 (86.7)	8 (72.7)		
Missing	3	3	NA		
Planned pregnancy					
Unplanned	2 (8.0)	1 (7.1)	1 (9.1)	0.03	0.99
Planned	23 (92.0)	13 (92.9)	10 (90.9)		
Missing	4	4	NA		

NA, Not Applicable. *p*-value was calculated on CHISQ test. When the cell size was smaller than 5, a Fisher exact test was applied. Bold indicates statistically significant.

**Table 2:** Drug-use-related characteristics among the study sample by self-perceived stage of recovery (n = 29).

	All (n = 29)	Low-Risk (n = 18)	High-Risk (n = 11)		*p*
	N (%)			χ^2^	
P30D drug use					
Opioids					
No	16 (69.6)	12 (92.3)	4 (40.0)	7.30	**0.02**
Yes	7 (30.4)	1 (7.7)	6 (60.0)		
Missing	6	5	1		
Tobacco					
No	7 (30.4)	7 (53.9)	NA	7.74	**0.01**
Yes	16 (69.6)	6 (46.2)	10 (100.0)		
Missing	6	5	1		
Cannabis					
No	14 (60.9)	9 (69.2)	5 (50.0)	0.88	0.42
Yes	9 (39.1)	4 (30.8)	5 (50.0)		
Missing	6	5	1		
Other (non-opioid) illicit drugs					
No	17 (77.3)	12 (100.0)	5 (50.0)	7.76	**0.01**
Yes	5 (22.7)	NA	5 (50.0)		
Missing	7	6	1		
Overdose					
Never	11 (45.8)	5 (35.7)	6 (60.0)	1.39	0.41
At some point of lifetime	13 (54.2)	9 (64.3)	4 (40.0)		
Missing	5	4	1		
Craving for opioids (Median, IQR)	4.0 (1, 19)	1.50 (1, 14)	12.0 (4, 20)	1.91 ^1^	0.06
Urine drug screen (Labs)					
i. MOUD					
Negative	5 (19.2)	1 (6.7)	4 (36.4)	3.60	0.13
Positive	21 (80.8)	14 (93.3)	7 (63.6)		
Missing	3	3	NA		
ii. Non-MOUD opioids (oxycodone, heroin, fentanyl, etc.)					
Negative	16 (61.5)	12 (80.0)	4 (36.4)	5.11	**0.04**
Positive	10 (38.5)	3 (20.0)	7 (63.6)		
Missing	3	3	NA		
iii. Other (barbiturates, benzos, cocaine, cannabis, etc.)					
Negative	13 (50.0)	10 (66.7)	3 (27.3)	3.94	0.11
Positive	13 (50.0)	5 (33.3)	8 (72.7)		
Missing	3	3	NA		
iv: ii + iii					
Negative	18 (69.2)	13 (86.7)	5 (45.5)	5.06	**0.04**
Positive	8 (30.8)	2 (13.3)	6 (54.5)		
Missing	3	3	NA		

NA, Not Applicable. P30D, Past 30-Day. IQR, Interquartile Range. *p*-value was calculated on CHISQ test. When the cell size is smaller than 5, a Fisher exact test will be applied. ^1^ The number indicates Z-score calculated on Mann-Whitey U test. Bold indicates statistically significant.

**Table 3:** Mental health characteristics among the study sample by self-perceived stage of recovery (n = 29).

	All (n = 29)	Low-Risk (n = 18)	High-Risk (n = 11)		*p*
	N (%)			χ^2^	
Mental health characteristics					
Depression					
None or mild	21 (72.4)	13 (72.2)	8 (72.7)	<0.001	0.99
Moderate or severe	8 (27.6)	5 (27.8)	3 (27.3)		
Anxiety					
None or mild	19 (65.5)	11 (61.1)	8 (72.7)	0.41	0.69
Moderate or severe	10 (35.5)	7 (38.9)	3 (27.3)		
Lifetime suicidal attempt					
No	16 (66.7)	9 (64.3)	7 (70.0)	0.09	0.99
Yes	8 (33.3)	5 (35.7)	3 (30.0)		
Missing	5	4	1		
P30D suicidal ideation					
No	19 (65.5)	13 (92.9)	10 (100.0)	0.75	0.99
Yes	10 (35.5)	1 (7.1)	NA		
Missing	5	4	1		

NA, Not Applicable. *p*-value was calculated on CHISQ test. When the cell size was smaller than 5, a Fisher exact test was applied.

## Data Availability

Data is unavailable due to privacy or ethical restrictions.

## Supplementary Materials

The following supporting information can be downloaded at: https://www.mdpi.com/article/10.3390/healthcare11172392/s1, Table S1: Summary of assessments.

## Appendix A. International Classification of Diseases, Tenth Revision (ICD-10) Used to Classify Participant Problems as Documented in the EPIC System

### Appendix A.1. Psychiatric Diagnoses

- Depression: F32
  F32.A unspecified
  F32.9 MDD, single episode, unspecified

- Anxiety: F41
  F41.1 Generalized Anxiety Disorder
  F41.9 anxiety disorder, unspecified

- Other mental disorder: F99

### Appendix A.2. Substance Use

- Methamphetamine: T43.65
  T43.651 unintentional poisoning
  T43.652 intentional self-harm

- Tobacco:
  Z72.0 Harmful Use
  F17.22 Nicotine Dependance, Chewing Tobacco
  F17.29 Nicotine Dependance, Other Tobacco Product

- Cocaine: F14
  F14.1 Cocaine Abuse
  F14.9 Cocaine Use, Unspecified

- Cannabinoid: F12
  F12.1 Cannabis Abuse
  F12.9 Cannabis Use, Unspecified

- Other psychoactive F19.1

### Appendix A.3. Medical Diagnoses

- Chronic Medical Conditions (examples)
  Asthma: J45-J46
  Atherosclerosis: I70
  Cancer: C00-C97
  Stroke: I60-I69
  Diabetes: E10-E14
  Heart Disease: I00-I109, I11, I13, I20-I51

- STI: A50-A64
  A64 Unspecified Sexually Transmitted Disease

- Obstetrics
  A09 Supervision of High-Risk Pregnancy
  O13 Gestational [pregnancy-induced] hypertension without significant proteinuria
  O16 Unspecified maternal hypertension
  O24 Diabetes mellitus in pregnancy
  ■ **O24.**1 Gestational diabetes mellitus
  O35 Maternal care for known or suspected fetal abnormality and damage
