# Supplementary material for: Self-Identified Stage in Recovery and Substance-Use Behaviors among Pregnant and Postpartum Women and People with Opioid Use Disorder
Source: Healthcare (Basel). 2023 Aug 25;11(17):2392. doi: 10.3390/healthcare11172392 (PMC10486579; doi:10.3390/healthcare11172392)
Supplement: Supplementary file 1 [file healthcare-11-02392-s001.zip › healthcare-2484406-supplementary.pdf]

Supplementary Table S1. Summary of assessments.

Variables of interest	Source	Question/Description	Responses
Self-perceived stage of recovery and risk level	Qualitative interview	Think about your recovery outside of your facility, at what stage in your recovery would you consider yourself to be	**High risk**: not engaged in treatment or a recovery program, new to recovery, or early in recovery vs. **Low risk**: well established or long-term recovery.
Socio-demographics			
Age	Baseline survey	How old are you?	**18-24** vs. **25-34** vs. **35 or above years old**
Race	Baseline survey	What ethnicity are you?	**Caucasian** vs. **African American**
Pregnant status	EPIC system	Number of weeks pregnant	**1st - 2nd trimester (week 0-26)** vs. **3rd trimester (week 27 or more) to delivered**
Marital Status	EPIC system	current marital status	**Single** vs. **Married** vs. **Other (divorced, widowed, separated)**
Type of MOUD	EPIC system	MAT type currently using	**Buprenorphine/Subutex/Suboxone** vs. **Others: Methadone/Naltrexone/Vivitrol/Others**
Education attainment	Baseline survey	What is the highest level of school you have completed?	**High school or below**: No schooling completed/1st -12th grade completed/high school completed/ GED certificate vs. **Some college or above**: some college credit, but no degree/ Associate's degree/ Bachelor’s degree/ Master's degree/ Doctorate degree/ Professional degree beyond a bachelor's degree
Insurance	Baseline survey	Are you covered by Medicaid or TRICARE, CHAMPUS, CHAMPVA, the VA, or military health care or private health insurance?	**Uninsured** vs. **Medicaid** vs. **Others**
Housing	Baseline survey	What is your current living arrangement?	**Unstable:** homeless (e.g., shelter, streets)/treatment facility/medical center vs. **Stable:** Living at someone else's home or apartment/Living in my own home/apartment
Employment	Baseline survey	Are you currently employed?	**No** vs. **Yes:** full-time/ part-time

Number of live births	EPIC system	Number of Live Births	**0-1** vs. **2 or more**
Father of baby involved	EPIC system	Father of Baby involved?	**No** vs. **Yes**
Planned pregnancy	EPIC system	Unplanned vs Planned Pregnancy	**Unplanned** vs. **Planned**
P30D drug use			
Opioids	Baseline survey	In the past month, how often have you used heroin/ methadone/ morphine/ OxyContin/ codeine/ fentanyl/ oxycodone/ hydrocodone/ hydromorphone/ other opioid analgesics or pain killers?	**No**: Not at all vs. **Yes**: A few times in the past month/A few times each week/Everyday
Tobacco	Baseline survey	In the past month, how often have you used tobacco?	**No**: Not at all vs. **Yes**: A few times in the past month/A few times each week/Everyday
Marijuana	Baseline survey	In the past month, how often have you used marijuana?	**No**: Not at all vs. **Yes**: A few times in the past month/A few times each week/Everyday
Other illicit drugs	Baseline survey	In the past month, how often have you used illicit drugs other than opioids/heroin?	**No**: Not at all vs. **Yes**: A few times in the past month/A few times each week/Everyday
Overdose	Baseline survey	How many times have you overdosed on drugs?	**Never** vs. **At some point of lifetime**: 1-3 times or more
Craving for opioids[1]	Baseline survey	How much do you currently crave opioids?	**0** (not at all) - **10** (extremely strong)
		In the past week, please rate how strong your desire to use opioids has been when something in the environment has reminded you of opioids.	**0** (not at all) - **10** (extremely strong)

		Please imagine yourself in the environment in which you previously used opioids. If you were in this environment today and if it were the time of day that you typically used opioids, what is the likelihood that you would use opioids today?	**0** (not at all) - **10** (extremely strong)
Urine drug screen (Labs)			
i. MAT	EPIC system		**Positive** vs. **Negative**
ii. Non-MAT opioids (oxycodone, heroin, fentanyl, etc.,)	EPIC system		**Positive** vs. **Negative**
ii. Other (barbiturates, benzos, cocaine, cannabis, etc.,)	EPIC system		**Positive** vs. **Negative**
iv: ii+iii	EPIC system	Non-MAT opioids (oxycodone, heroin, fentanyl, etc.) & others (barbiturates, benzos, cocaine, cannabis, etc.)	**Positive** vs. **Negative**
Mental health characteristics			
Depression (assessed by PHQ-9)[2]	Baseline survey	Over the last 2 weeks, how often have you been bothered by any of the following problems?	
		Little interest or pleasure in doing things	**0** (not at all) - **3** (nearly everyday)
		Feeling down, depressed, or hopeless	**0** (not at all) - **3** (nearly everyday)
		Trouble falling or staying asleep, or sleeping too much	**0** (not at all) - **3** (nearly everyday)
		Feeling tired or having little energy	**0** (not at all) - **3** (nearly everyday)

		Poor appetite or overeating	**0** (not at all) - **3** (nearly everyday)
		Feeling bad about yourself-- or that you are a failure or have let yourself or your family down	**0** (not at all) - **3** (nearly everyday)
		Trouble concentrating on things, such as reading the newspaper or watching television	**0** (not at all) - **3** (nearly everyday)
		Moving or speaking so slowly that other people could have noticed or the opposite--- being so fidgety or restless that you have been moving around a lot more than usual	**0** (not at all) - **3** (nearly everyday)
		Thoughts that you would be better off dead or of hurting yourself in some way	**0** (not at all) - **3** (nearly everyday)
Anxiety (assessed by GAD-7)[2]	Baseline survey	Over the last 2 weeks, how often have you been bothered by any of the following problems?	
		Feeling nervous, anxious, or on edge	**0** (not at all) - **3** (nearly everyday)
		Not being able to stop or control worrying	**0** (not at all) - **3** (nearly everyday)
		Worrying too much about different things	**0** (not at all) - **3** (nearly everyday)
		Trouble relaxing	**0** (not at all) - **3** (nearly everyday)
		Being so restless that it's hard to sit still	**0** (not at all) - **3** (nearly everyday)
		Becoming easily annoyed or irritable	**0** (not at all) - **3** (nearly everyday)

		Feeling afraid as if something awful might happen	**0** (not at all) - **3** (nearly everyday)
Lifetime suicidal attempt	Baseline survey	Have you ever, in your whole life, tried to kill yourself or made a suicide attempt?	**No** vs. **Yes**
P30D suicidal ideation	Baseline survey	Has there been a time in the past month when you have had serious thoughts about ending your life?	**No** vs. **Yes**
Current diagnoses from EPIC			
1. Psychiatric (non-SUD)	EPIC system	See Appendix 1.	**No** vs. **Yes**
2. Substance Use Disorder (SUD)	EPIC system	See Appendix 1.	**No** vs. **Yes**
3. Medical	EPIC system		
Chronic Medical Condition	EPIC system	See Appendix 1.	**No** vs. **Yes**
Obstetric Diagnosis:	EPIC system	See Appendix 1.	**No** vs. **Yes**
STI/STD[4]	EPIC system	See Appendix 1.	**No** vs. **Yes**

[1] A continuous variable was created to assess self-reported craving to opioids by using a sum of 3 items. The possible scale scores range from 0 to 30, with higher scores indicate the stronger craving.

[2] A cutoff of 9 was applied to dichotomize as none or mild vs. moderate or above depression/anxiety.
